# P accumulation and physiological responses to different high P regimes in *Polygonum hydropiper* for understanding a P-phytoremediation strategy

**DOI:** 10.1038/srep17835

**Published:** 2015-12-09

**Authors:** Daihua Ye, Tingxuan Li, Dan Liu, Xizhou Zhang, Zicheng Zheng

**Affiliations:** 1 College of Resources, Sichuan Agricultural University, Chengdu 611130, Sichuan, China

## Abstract

Phosphorus (P) accumulators used for phytoremediation vary in their potential to acquire P from different high P regimes. Growth and P accumulation in *Polygonum hydropiper* were both dependent on an increasing level of IHP (1–8 mM P) and on a prolonged growth period (3-9 weeks), and those of the mining ecotype (ME) were higher than the non-mining ecotype (NME). Biomass increments in root, stem, and leaf of both ecotypes were significantly greater in IHP relative to other organic P (Po) sources (G1P, AMP, ATP), but lower than those in inorganic P (Pi) treatment (KH_2_PO_4_). P accumulation in the ME exceeded the NME from different P regimes. The ME demonstrated higher root activity compared to the NME grown in various P sources. Acid phosphatase (Apase) and phytase activities in root extracts of both ecotypes grown in IHP were comparable to that in Pi, or even higher in IHP. Higher secreted Apase and phytase activities were detected in the ME treated with different P sources relative to the NME. Therefore, the ME demonstrates higher P-uptake efficiency and it is a potential material for phytoextraction from P contaminated areas, irrespective of Pi or Po contamination.

Soil phosphorus (P) is an important nutrient source yet least available to plant growth^1^. Inorganic P (Pi) is the major fraction available for plants to acquire from soil. However, abundant P exists as organic P (Po), including phosphomonoesters, phosphate diesters and sugar phosphates^2^,^3^. Soil Po is regarded as a major potential source to plant growth^4^. In order to utilize and uptake P from soil Po, plants have formed a series of physiological adaptations. Production and secretion of acid phosphatase (Apase) and phytase are vital physiological mechanisms that promote the potentials of P acquisition and regulate plant P nutrition^5^,^6^,^7^,^8^. Apase is a kind of non-specific phosphatase catalyzing the mineralization of Po to yield available Pi^9^. Phytase, a specialized enzyme, is of particular interest due to its ability to realize the hydrolysis of inositol pentakisphosphate and hexakisphosphate (phytate) which constitutes up to 80% of total Po in soil^2^,^6^,^10^.

Previous studies showed that abundant Po existed in animal manure. He *et al*. found that poultry litter contained 40%–70% phytate-like P and 10%–30% simple monoester P in Po fractions extracted by hydroxide and acid^11^. The hydrolyzable Po fraction for various manures containing monoester-, phytate-, and DNA-like P was dominated by phytate- and monoester-like P, particularly in chicken and swine manure^12^. Agricultural land usage is a common fate of the large amounts of animal manure to supply nutrients for plant growth and improve nutrient recycling^12^. Thus, a large quantity of undigested feed Po is excreted and put into farmland with animal manure^13^. Repeated and substantial application of animal manure to farmland will increase risks of accumulation of Po in soils and P-pollution due to P runoff and leaching. Therefore, more attention should be paid to Po in potential environmental P-pollution issues.

P remediation using plants has been certified as an effective means to remove P from P-polluted soils. Several vegetable species (e.g. *Cucurbita pepo* var. *melopepo*, *Cucumis sativus*) and some grasses (e.g. *Lolium multiflorum* L., *Duo festulolium*) have been reported as potential P accumulators for their shoot P >1% dry weight (DW) when grown in high P conditions^6^,^13^,^14^,^15^,^16^. However, these P accumulators have defects like; relatively low DW yield, low P accumulation potential, or are not adaptive to polluted water. *Polygonum hydropiper* represents a worthy candidate to remediate excess P because of its great attributes of being able to grow in both terrestrial and aquatic areas, and high potentials of P uptake and P removal.

A mining ecotype (ME) and a non-mining ecotype (NME) of *P. hydropiper* grown in high P soil or hydroponic media yielded great DW and demonstrated high shoot P accumulation, and the ME accumulated significantly higher P than the NME when Po existed in the growth media^17^,^18^,^19^,^20^. P fractions characteristics and rhizosphere processes in both ecotypes of *P. hydropiper* have preliminarily been investigated in soils amended with swine manure, and the ME was more effective in obtaining P from Po fractions compared to the NME^21^. The previous studies provide a sound theoretical basis for evolving a P-phytoextraction strategy in the ME and NME grown in sole Po conditions. In the above backdrop, it is necessary to achieve a thorough understanding of the pattern of P nutrition in the two ecotypes using Po sources. In this study, we supposed that the ME and the NME may differ in P uptake from Po and their physiological responses to Po supply remained different. Therefore, three different experiments were performed to compare the differences between the ME and the NME in: 1) tolerance by determining biomass under high levels of P or different growth periods; 2) P uptake ability by analyzing tissue P accumulation; 3) physiological responses by determining root activity, extracted Apase and phytase activities, and secreted Apase and phytase activities from the roots to assess the utilization of Po when the two ecotypes of *P. hydropiper* were grown in a range of media supplied with different P sources.

## Results

### Biomass and P accumulation of *P. hydropiper* grown under different high Po levels

**Figure 1:**
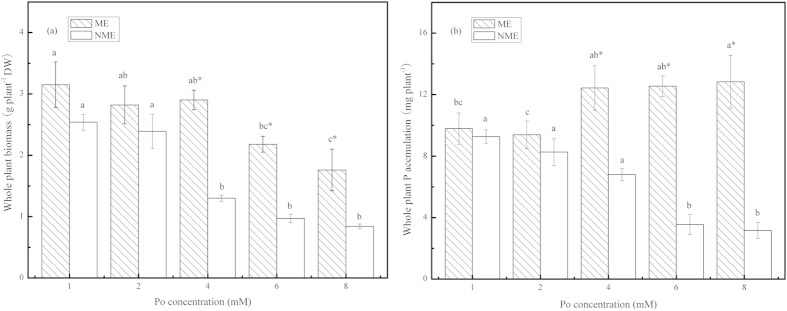
Biomass (**a**) and P accumulation (**b**) in the whole plant of *P. hydropiper* grown under hydroponic media containing 1–8 mM P supplied as IHP for 5 weeks. ME, mining ecotype; NME, non-mining ecotype. Values represent mean ± standard error of four replicates. The histograms with different small letters are statistically different (*p* < 0.05) among the various Po concentrations, and * represents significant difference (*p* < 0.05) between the two ecotypes.

Biomass in the whole plant of *P. hydropiper* grown in hydroponic media supplied with a series of Po levels up to 8 mM is shown in Fig. 1a. Whole plant biomass significantly reduced in both ecotypes of *P. hydropiper*. A significant decrease in whole plant biomass was observed at 6 mM and 4 mM for the ME and the NME, respectively. Biomass in the ME was significantly higher compared to the NME when both were grown in high *myo*-inositol hexaphosphoric acid dodecasodium salt (IHP) media at 4, 6 and 8 mM.

*P. hydropiper* accumulated different P amounts in the whole plant when grown in different levels of Po added as IHP (Fig. 1b). Whole plant P accumulation of the ME significantly increased at 4 mM, beyond which a stable trend was noticed. However, a continued slowdown of whole plant P accumulation was observed in the NME with the increasing Po concentrations. Whole plant P accumulation in the ME was significantly greater relative to that of the NME. The ME demonstrated whole plant P accumulation in a relatively narrow range of 9.39–12.84 mg plant^−1^, while the NME’s whole plant P accumulation ranged from 3.16–9.27 mg plant^−1^.

### Biomass and P accumulation of *P. hydropiper* grown at different growth periods

As shown in Table 1, biomass of both ecotypes significantly increased with the increasing growth periods. The greatest increment of biomass was observed from 5 to 7 weeks in both ecotypes. The ME showed significantly greater biomass in root, stem, and leaf relative to the NME at 7 weeks and there were no obvious differences in the biomass between the two ecotypes in the other growth periods. This suggests that both ecotypes are able to obtain P from high concentrations of IHP.

P accumulations in the two ecotypes differed greatly among the different growth periods (Table 2). P accumulations of both ecotypes were in the order of stem>leaf>root. Stem and leaf P accumulations of both ecotypes significantly increased with prolonged growth periods. Stem P accumulation in the ME seedlings increased in response to increasing IHP and it reached 31.07 and 40.94 mg plant^−1^ at 7 and 9 weeks, respectively. The same pattern was noticed in leaf P accumulation. In addition, the ME demonstrated significantly higher P accumulations in the stem and leaf compared to the NME.

### Biomass and P accumulation of *P. hydropiper* grown under different P sources

**Figure 2:**
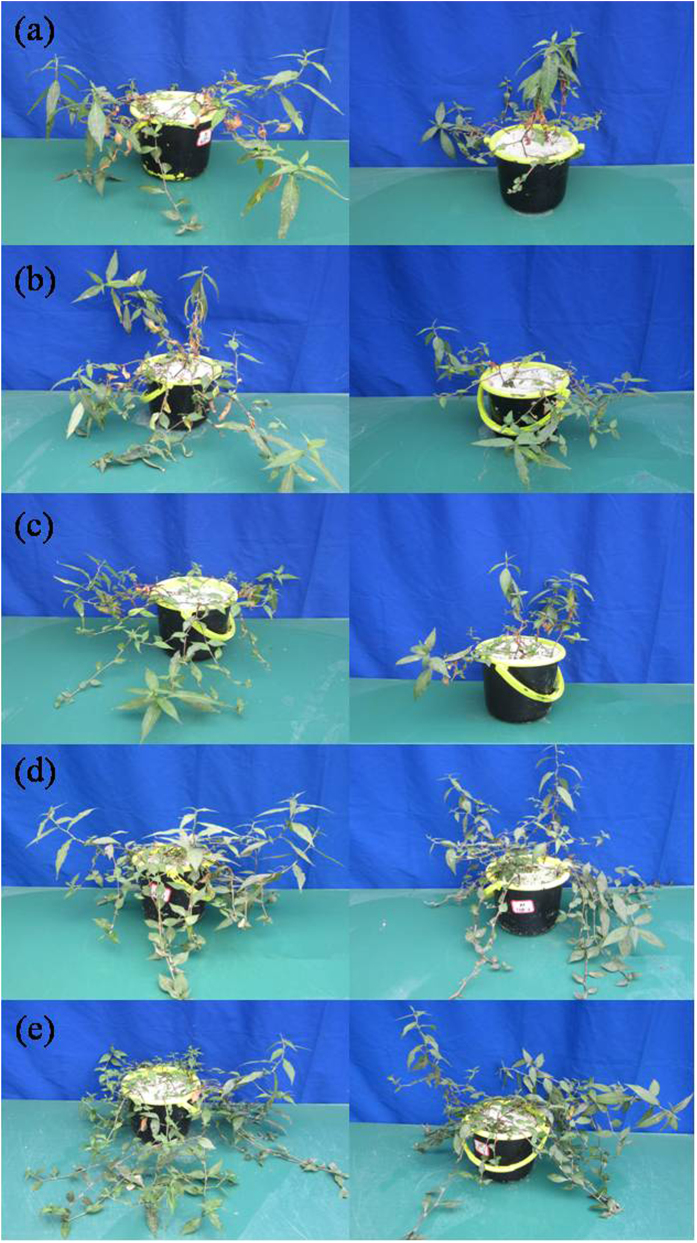
The growth of the mining ecotype (left) and the non-mining ecotype (right) of *P. hydropiper* grown under perlite media containing 3 mM P supplied either as G1P (**a**), AMP (**b**), ATP (**c**), IHP (**d**), or Pi (**e**) for 5 weeks.

Plants grown in G1P, AMP, and ATP showed stunted growth with small, yellow leaves and lesser stems. However, there were no symptoms of P toxicity on the seedlings grown in IHP and Pi (Fig. 2). Both ecotypes of *P. hydropiper* demonstrated different biomass when grown in various P sources (Table 3). In both ecotypes, maximum biomass in root, stem, and leaf was noticed in Pi media. Biomass of ME and NME grown in IHP was significantly higher than that in other Po sources. In addition, the ME registered significantly greater stem DW in G1P and AMP than the NME. There were no significant differences in leaf DW between the two ecotypes.

In both ecotypes, P accumulations were dependent on both P sources and ecotypes (Table 4). The ME accumulated P in the roots from various P sources ranging from 0.50–2.47 mg plant^−1^, which was significantly greater than root P accumulation of the NME in G1P and AMP. The seedlings exhibited a similar pattern with a highest stem P accumulation of 29.76 mg plant^−1^ for the ME and 22.82 mg plant^−1^ for the NME from Pi media, followed by accumulation from IHP media. In addition, the ME showed a significantly higher stem P accumulation of 1.30–1.93 times compared to the NME, irrespective of P sources. Leaf P accumulation from IHP source was comparable to P amount accumulated from Pi source. The accumulations from IHP and Pi media were significantly greater than from the other Po sources in both ecotypes. No significant difference was noticed in accumulations of leaves in the ME and NME even in Pi media, except for the media supplied with AMP.

### Root activity of *P. hydropiper* grown under different P sources

**Figure 3:**
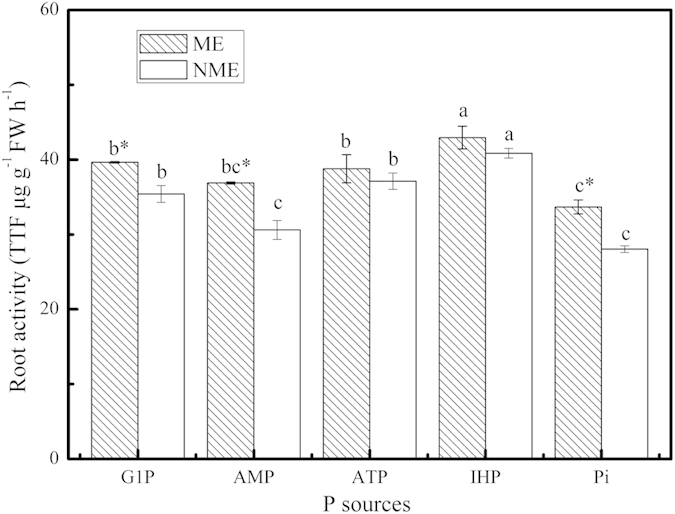
Root activity of *P. hydropiper* grown under perlite media containing 3 mM P supplied either as G1P, AMP, ATP, IHP, or Pi for 5 weeks. ME, mining ecotype; NME, non-mining ecotype. Values represent mean ± standard error of six replicates. The histograms with different small letters are significantly different (*p* < 0.05) among P sources. * indicates significantly different (*p* < 0.05) between ecotypes.

*P. hydropiper* showed variable root activity depending on growth media and plant ecotypes (Fig. 3). Root activity ranged between 33.67 and 42.94 TTF μg g^−1^ FW h^−1^ for the ME and from 28.04 to 40.86 TTF μg g^−1^ FW h^−1^ for the NME, respectively. In both ecotypes, root activity reached a maximum when grown in the presence of IHP while an interesting phenomenon that the Pi growth media caused a lower root activity than the other P sources was observed. The ME exhibited on average 12% greater root activity in comparison to the NME and significantly higher than the NME grown in the presence of G1P, AMP, and Pi.

### Activities of APase and phytase in root extracts of *P. hydropiper* grown under different P sources

**Figure 4:**
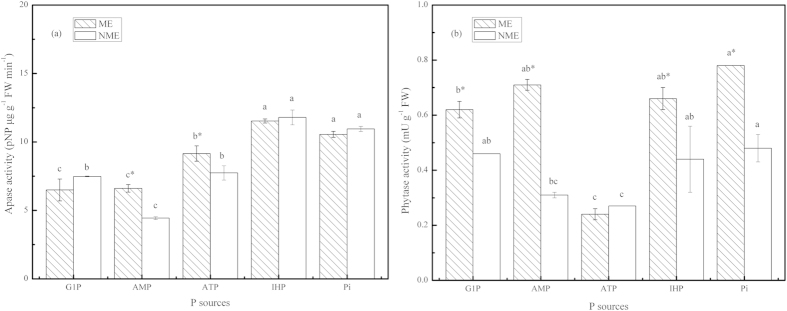
Activities of APase (**a**) and phytase (**b**) in root extracts of *P. hydropiper* grown under perlite media containing 3 mM P supplied either as G1P, AMP, ATP, IHP, or Pi for 5 weeks. ME, mining ecotype; NME, non-mining ecotype. Values represent mean ± standard error of six replicates. The histograms with different small letters are significantly different (*p* < 0.05) among P sources. * indicates significantly different (*p* < 0.05) between ecotypes.

The pattern of Apase and phytase in the root extracts of the two ecotypes are displayed in Fig. 4. Greatest Apase activity among the P-fed plants was observed in the root extracts treated with IHP, followed closely by Pi, ATP, G1P, and AMP treatments. An activity of 11.53 and 11.79 pNP μg g^−1^ FW min^−1^ was determined in the roots of the ME and NME seedlings supplied with IHP media, respectively. The ME showed significantly greater Apase activity in the root extracts compared to the NME in AMP and ATP. Phytase activity in the roots treated with various P sources ranged between 0.27 and 0.78 mU g^−1^ FW. Both ecotypes grown in the presence of ATP demonstrated significantly lower root phytase activity. Phytase activity in the root extracts of the ME was significantly greater by 35%–129% than that of the NME in any P source treatment, excluding ATP treatment.

### Activities of APase and phytase in root secretions of *P. hydropiper* grown under different P sources

**Figure 5:**
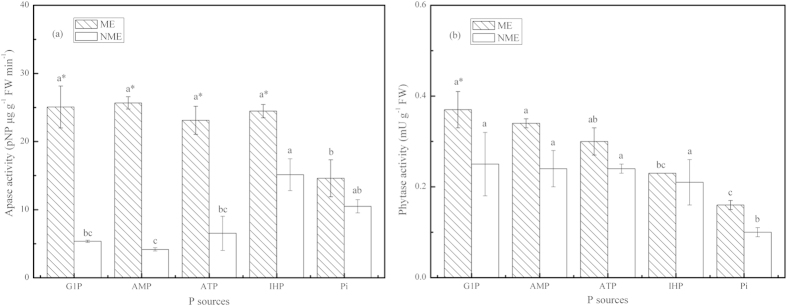
Secreted APase (**a**) and phytase (**b**) activities of *P. hydropiper* grown under perlite media containing 3 mM P supplied either as G1P, AMP, ATP, IHP, or Pi for 5 weeks. ME, mining ecotype; NME, non-mining ecotype. Values represent mean ± standard error of six replicates. The histograms with different small letters are significantly different (*p* < 0.05) among P sources. * indicates significantly different (*p* < 0.05) between ecotypes.

Root secreted APase and phytase activities were analyzed in the 5 week-old seedlings of *P. hydropiper* cultured in different P sources (Fig. 5). Apase activity in the root secretions varied between 14.60 and 25.67 pNP μg g^−1^ FW min^−1^ from the ME and between 4.16 and 15.13 pNP μg g^−1^ FW min^−1^ from the NME treated with various P sources. Apase activity was significantly enhanced in the ME seedlings cultured in the Po media compared to Pi media. Highest Apase enzyme activity was observed in root secretions of the NME seedlings grown in the presence of IHP. In both ecotypes, statistical differences were detected in Apase activity between the ME and NME grown in various P sources, except for the Pi treatment. Po sources significantly increased phytase activity in both ecotypes compared to Pi source, and the ME secreted more phytase relative to the NME under different P sources.

## Discussion

### Growth and P uptake of *P. hydropiper*

The results in hydroponic experiment revealed growth in both ecotypes associated with increasing levels of IHP (1–8 mM P) (Fig. 1a) and different growth periods (Table 1). The ME demonstrated better tolerance and adaptability than the NME grown in high IHP media with 4, 6, and 8 mM P, resulting in its significantly greater biomass. The second hydroponic experiment confirmed this once more. The ME was more tolerant compared to the NME, generating a significant difference in biomass with the growth period prolonged. In addition, we attempted to investigate the potential role of the two ecotypes of *P. hydropiper* to assimilate and extract P from perlite media supplied with various P sources including monoester P, diester P, phytate-P, and Pi. Both ecotypes of *P. hydropiper* registered high DW yield when grown in the media with different P sources. The biomass in wild-type seedlings of *Arabidopsis*^22^, *Trifolium subterraneum* L.^23^, and *Solanum*^24^ significantly decreased when grown under Po media, particularly IHP. However, the capability of utilizing Po from various P sources in some P accumulators (e.g. *D. festulolium* and two cultivars of annual ryegrass) to meet their growth was great^13^,^16^. Tissue biomass of Gulf ryegrass showed significant decrease when seedlings were grown in monoester (G1P, ATP) and diester P (AMP) media compared to IHP and Pi^16^. A similar case was observed in our study (Table 3). Growth pattern of both ecotypes in the three experiments suggested that seedlings can acquire P from Po media, particularly IHP, as effectively as from Pi to realize an optimal growth. In addition, the ME registered greater tissue biomass and root activity than the NME in different high Po regimes, suggesting that the ME of *P. hydropiper* demonstrated high tolerance in P-rich media and suffered less P toxicity. Results from this study agreed with our previous researches that reported better growth in the ME than the NME when grown under high P conditions^8^,^18^,^19^.

The data from the three experiments showed that *P. hydropiper* was capable of assimilating more P from high levels of different P media, particularly from IHP and Pi media, and mainly accumulated it in their shoots (Fig. 1b, Tables 2 and 4). Tissue P content (data not presented) of both ecotypes grown in high P regimes reached a level lower than that recorded for the newly reported Australian native genera (*Ptilotus polystachyus*)^25^, and even lower than Duo grass and annual ryegrass grown in media supplied with the same P sources^13^,^16^. As a plant used for phytoextraction, it should be able to tolerate a high concentration of the pollutant and accumulate great amounts of the target element in the harvested part^26^,^27^. A significant increase in biomass of *P. hydropiper* was noticed compared to Duo grass and ryegrass of which biomass did not reach 60 mg DW for 2 weeks growth^13^,^16^. This attribute of *P. hydropiper* compensates for the relatively low tissue P content, and P uptake potential of *P. hydropiper* is more promising. In addition, the earlier researches reported that P acquisition ability from IHP in some plants was limited^4^,^22^. Wheat (*T. subterraneum*) showed poor utilization from IHP and exhibited 74.4 μg P in the shoots, just 20% of P accumulation in seedlings grown in Pi media^4^. Richardson *et al*. reported *Arabidopsis* seedlings showed significantly lower P assimilation directly from IHP than from Pi media^22^. In both sterile and non-sterile soil conditions, *Triticum aestivum* L. also showed limited ability to utilize P from phytate^28^. However, expression of phytase gene in transgenic lines significantly enhanced plant DW and P uptake when grown in the sole source of P supplied as phytate compared to the wild-type seedlings^22^,^29^. The transgenic lines with high-expressing Apase and phytase genes had the potential to acquire more P and yield higher biomass^30^. Giles *et al*.^31^ reported that *Nicotiana tabacum* inoculation with *Pseudomonas sp. CCAR59*, an organic anion-producing and phytate-hydrolyzing soil isolated bacteria, enhanced 6-fold shoot P accumulation of wild-type plants when grown on calcium-IHP. In our study, P uptake from IHP in both ecotypes of *P. hydropiper* surpassed the above common plant species, with it well comparable to transgenic lines of expressing Apase or phytase gene, or plants colonized with a phytase- or organic anion-producing microorganisms^22^,^23^,^24^,^30^,^31^,^32^,^33^,^34^,^35^. Whole plant P accumulation in the ME did not decrease with increasing Po levels, suggesting that *P. hydropiper* might be effectively used for IHP removal from eutrophic water with different degrees of pollution. Furthermore, an interesting result observed in the two ecotypes was that higher biomass and P content was accumulated in the ME seedlings supplied with any P level or P source. Thus, greater shoot P accumulation in the ME was observed in high P media, indicating the ME was more efficient relative to the NME and other P accumulators to uptake and remove P, and it is a promising species for phytoremediation of P polluted areas.

Plant growth and P uptake were affected by growth period^26^. Waldrip *et al*.^36^ reported that both root and shoot P uptake of *Lolium perenne* L. were significantly greater at 16 weeks than 8 weeks when grown under high P conditions. Extending the growth period was directly responsible for a higher P removal of *L. multiflorum* from eutrophic water^37^. Therefore, the harvest time also affected the capacity of P removal by P accumulators from real eutrophic water or high P soils. It will significantly improve phytoremediation efficiency by harvesting seedlings at a growth period with the maximum ability for P uptake. In this study, P accumulation was dependent on the growth period and reached a maximal value in 9 weeks. In addition, seedlings was just in flowering stage of 9-week growth and an advisable measure involving harvesting of *P. hydropiper* in flowering might be effective in decreasing excess P levels.

### Root physiological characteristics in response **to various P sources**

Po is a potential and important nutrient pool for plant growth, and it requires phosphatases to be mineralized to promote plant P nutrition^38^,^39^. In the present study, phosphatases like Apase and phytase in root extracts and secretions of the two ecotypes of *P. hydropiper* were investigated in perlite media supplied with different P sources. As presented in Figs 4 and 5, it clearly revealed that both ecotypes demonstrated the potential to produce and secrete a higher amount of Apase in response to various P sources, compared to swine manure treatment^19^. Higher Apase activity was observed in root extracts of both ecotypes grown in the presence of IHP compared to seedlings in other Po sources and Pi media. A similar trend was observed in ryegrass, but was completely in disaccord with Duo grass and *Arabidopsis* grown in these P sources^13^,^22^. Obviously, greater Apase activity was determined in root secretions than in root extracts, regardless of P sources (Figs 4 and 5). The enhanced secretion of Apase into perlite media promotes plant P uptake from Po sources as Apase shows nonspecific activity towards various Po. The ME had a relatively limited ability to acquire P from G1P, AMP, and ATP compared to IHP even though high Apase activity was also detected both in root extracts and secretions. A contrary report showed that a decreasing Apase activity was negatively related to the increased P acquisition^16^. AMP, belonging to diester-P, should be catalyzed by phosphodiesterases. There might be slight phosphodiesterases in the present study and this hypothesis needs further study to verify. The two ecotypes showed no significant differences in root extracted and secreted APase when grown in Pi media, however, the ME accumulated notablely higher P in stem compared to NME, a similar pattern also reported in our previous study^8^. *D. festulolium* demonstrated significantly higher Apase activity than *L. multiflorum*, however, its shoot P content was less than *L. multiflorum* when grown in Po substrates^13^,^16^. Thus, integrated attributes are the key decisive factors to heighten P assimilation in a plant. Furthermore, it was also observed that extracted and secreted Apase activity of the ME was significantly higher than that of the NME as a result of more appreciable tissue biomass and P uptake.

Production and secretion of phytase is another root physiological characteristic in many plant species to acquire P from phytate-P^13^,^22^. Phytase, a phosphomonoesterase, shows highly specific affinity for phytate^2^,^15^. Some plant species have been reported exhibiting poor ability to obtain P from IHP most likely due to low activity of phytase. The utilization of P from IHP in *Arabidopsis* and *T. subterraneum* is connected in scanty intrinsic or extracellular phytase^22^,^23^. The inability of *T. aestivum* L. to use phytate was not because of poor availability of substrates but due to deficient activities of phytase^22^,^23^. However, *D. festulolium* and *L. multiflorum* were capable of producing and secreting sufficient enzymes of phytase to meet with different P sources^13^,^16^, with which our study was in agreement for the ME. From all data, it was obvious that although phytase activity was equivalent to a small part of total Apase activity in both ecotypes, *P. hydropiper* exhibits superior efficiency in IHP acquisition compared with most reported species cases. Recently, some plants were confirmed unable to utilize phytate to improve P accumulation in spite of being provided with the phytate gene^16^. George *et al*. reported that seedlings expressing phytate gene (*phyA*) did not show greater P accumulation than control seedlings^40^. Thus, poor availability of phytate in environments and the insufficient phytase activity in root productions and secretions are two major factors that limit plant improving P-nutrition from phytate. Root phytase plays a critical role in P nutrition from phytate due to its abundant secretions to reach substrates^16^. Unlike the pattern of Apase, the secreted phytase activity was lower than the enzyme activities in the root extracts. Thus, we hypothesized that enhanced secreted phytase activities of *P. hydropiper* by development of transgenics or inoculation of a phytase-producing microorganism might particularly work to improve P uptake from IHP and reduce the risk of animal manure usage with diminishing Po pollution. In addition, the ME demonstrated higher phytase activity in both extracts and secretions compared to the NME. Linked with our previous reports^8^,^18^,^19^, high extracellular activities of Apase and phytase from roots, not just high levels of intracellular Apase and phytase activities, are key stimulators to P utilization and uptake of *P. hydropiper* from various P sources.

## Materials and Methods

### Plant material

Seedlings of a mining ecotype (ME) and a non-mining ecotype (NME) of *P. hydropiper* were obtained from a P-mine site in Shifang (104°50′ E, 30°25′ N) and an uncontaminated agricultural area in Ya’an (102°59′ E, 29°59′ N), respectively, in May of 2013 and 2014. Healthy and uniform seedlings were selected and pre-cultured on vermiculite for 1 week with 1:10 Hoagland’s solution^18^.

### Growth and harvest of *P. hydropiper*

Modified Hoagland’s salts mixture without monopotassium phosphate (KH_2_PO_4_) as described by Ye *et al*.^8^ was used as basal nutrient medium. There were three pot experiments as follows:

In 2013, the first hydroponic experiment was carried out to determine the effect of increasing levels of Po (1, 2, 4, 6, and 8 mM) added as IHP [Sigma] on the growth and P uptake of *P. hydropiper*. Four replicates were performed for each treatment. To prevent light diffusion into the root, the pots were painted outside with a black varnish. Two healthy and uniform plants were transferred in each pot with 5 L of modified Hoagland’s solution. The seedlings were fixed by the sponge and hard cystosepiment to keep the shoots above cystosepiment. The cystosepiment has two apertures of 2 cm and its thick is 2 cm. The nutrient media (pH 5.8) were replaced every 5 days. Plants were harvested after 5 weeks of growth with sunlight. Plants were gently removed from the cystosepiments. Subsequently, samples were washed with tap water and distilled water, respectively and blotted with absorbing paper.

In 2014, the second hydroponic experiment was performed in a greenhouse using barrels of 3.5 L filled with basal media with 3 mM P supplied as IHP. The pretreatment of the corresponding items was the same as the above mentioned. Four healthy seedlings of similar size were transferred to 1:2 Hoagland’s solution. Seedlings of 10 d old were then transplanted into nutrient solution with 3 mM P added as IHP. Experimental treatment was repeated using three replicates for each harvest. Barrels were randomized by a complete block design. Solutions were changed every 4 or 5 days. This experiment was performed with sunlight. Pot plants were harvested at 3, 5, 7 and 9 weeks after transplantation, respectively. The harvested plants were treated as above and divided into root, stem and leaf.

In 2014, the third experiment was conducted to investigate the effect of various Po sources on P accumulation and physiological characteristics of *P. hydropiper*. Perlite was selected as the immobilizing matrix. Basal nutrient solution medium (pH 5.8) was used by adding 3 mM P supplemented either as α-D-glucose 1-phosphate disodium salt (G1P), adenosine 3′:5′ cyclic monophosphate sodium salt (AMP), adenosine-5′-triphosphate disodium salt (ATP), or IHP [Sigma]. The control was 3 mM P added as KH_2_PO_4_ (Pi). Four healthy and uniform seedlings of *P. hydropiper* were first transferred to 1:2 Hoagland’s solution for preculture of 10 days, and then transplanted respectively in each barrel (3.5 L) containing 0.3 kg perlite and 2 L of the medium. Further addition of the medium was 300 mL every 3 or 4 days for each pot. To compensate the evaporation, water management was performed by the weight method. This greenhouse experimental design was a completely randomized design and each treatment was replicated six times. After 5 weeks of growth with sunlight, the plants were harvested. They were treated as above and divided into root, stem and leaf.

### Analysis of root activity

Roots from different P regimes were excised after harvesting. The method proposed by Xiong^41^ was modified as follows: about 0.15 g fresh weight (FW) of intact seedling roots were immersed in a 50 mL triangular flask containing 5 mL of 0.4% triphenyltetrazolium chloride (TTC) and 5 mL phosphate buffer. The reaction was maintained at 37 °C and incubated for 2 h. After incubation, 2 mL of 1 M sulphuric acid solution was added to stop reaction. A control was determined by adding 2 mL of 1 M sulphuric acid solution first, then adding fresh roots. The other operating steps in the control test were done according to the above procedures. After reaction, all roots were taken out from the triangular flask, and dried using absorbent paper. The dried roots were homogenized with a mortar and pestle in 2 mL acetic ether and the supernatants were transferred into a 10 mL volumetric flask. The extraction process was repeated 2–3 times until the supernatant was colourless. The absorbance of the colored solution was determined spectrophotometrically at 485 nm. In this method, when TTC contacts with live cells of roots, it will be reduced by dehydrogenase enzymes into triphenyltetrazolium formazan (TTF). Thus, the colorless root will turn to red root, and shades of red in the roots are positively correlated with root activity. Root activity was measured from the release of TTF and defined as TTF μg g^−1^ FW h^−1^.

### Determination of P content

The preparation and dissolution of the samples from the three experiments was done according to the method of Ye *et al*.^19^. Tissue P content was analyzed colorimetrically at 700 nm using a UV-VIS spectrophotometer^42^.

### Apase and phytase activities in root extracts

Roots from different P regimes were separated after being washed thoroughly, then frozen in liquid nitrogen and stored at −80 °C. Fresh tissues of 0.3 g were chilled on ice, and homogenized with a mortar and pestle in 5 mL of 15 mM 2-morpholinoethanesulfonic acid, monohydrate (MES) buffer (0.5 mM CaCl_2_·H_2_O, 1 mM EDTA, and pH 5.5). The extracts were centrifuged at 4 °C (10,000 rpm, 20 min) and the supernatants were used to analyze activities of Apase and phytase.

Apase and phytase activities were performed using p-nitrophenyl phosphate disodium salt hexahydrate (pNPP) and IHP as substrates, respectively, based on the methods of earlier reports^10^,^15^. The assay for APase activity was performed in 3 mL liquid containing 2 mL of 10 mM pNPP, and 1 mL enzyme extract. The components were mixed and Apase activity was determined after 30 min incubation at 37 °C followed by the addition of 2 mL 0.25 M NaOH. The reaction for analysis of phytase activity was initiated by the addition of 2 mL of 15 mM MES buffer (pH 5.5) to an assay mixture containing 1 mL enzyme extract and 1 mL of 2 mM IHP. 2 mL of ice-cold 20% (w/v) trichloroacetic acid was added to terminate the reaction after incubation at 37 °C for 60 min. APase and phytase activities were determined from the release of p-nitrophenol (pNP) and soluble Pi spectrophotometrically using a UV-VIS spectrophotometer at 412 nm and 882 nm, respectively. Thus, APase activity was defined as pNP μg g^−1^ FW min^−1^. Phytase activity was expressed as mU g^−1^ root FW, where 1 U releases 1 μmol of soluble Pi min^−1^.

### Apase and phytase activities in root secretions

Five week-old seedlings grown in the various P media were harvested and the roots were washed with sterile deionized water and wiped. The roots were incubated for 2 h in 30 mL of 15 mM MES buffer (pH 5.5) containing 10 mM pNPP for APase or 2 mM IHP for phytase. For phytase, roots were also incubated in buffer in the absence of IHP as a control to account for the possible P efflux from roots. The roots were washed with distilled water after the incubation, and the FW was recorded. The secreted APase and phytase activities were analyzed as described above.

### Statistical analysis

Statistical analyses were conducted by the DPS 11.0 software package using variance analysis. Differences at significant level of *p* < 0.05 were estimated using LSD. Graphical work was accomplished by Origin 8.0.

## Conclusions

Different plant ecotypes responded differently to P media, depending on the supplied P levels, growth periods and P sources. The ME was more tolerant to high levels of Po (IHP) and therefore demonstrated higher biomass and P accumulation. Superior capability in growth and P uptake was also observed in the ME from various Po sources, particularly IHP, compared to that in the NME. G1P, ATP, and AMP reduced the biomass and P accumulation in both ecotypes grown in perlite media relative to IHP and Pi. In addition, higher root activity, APase and phytase activities in root extracts and secretions were observed in the ME compared with the NME. Thus, enhanced root productions and secretions of Apase and phytase may be responsible for increased mineralization of various Po sources to release available P for ME seedlings growth and P uptake. It thus reveals that the ME has an efficient P uptake mechanism in response to Po sources and has attractive potential of extracting P from P-polluted area.

## Additional Information

**How to cite this article**: Ye, D. *et al*. P accumulation and physiological responses to different high P regimes in *Polygonum hydropiper* for understanding a P-phytoremediation strategy. *Sci. Rep*. **5**, 17835; doi: 10.1038/srep17835 (2015).

## Figures and Tables

**Table 1:** Biomass of *P. hydropiper* grown under 3 mM P supplied as IHP for different growth periods (g plant^−1^ DW).

Growth period(weeks)	Root		Stem		Leaf	
	ME	NME	ME	NME	ME	NME
3	0.13 ± 0.01c	0.12 ± 0.01c	0.83 ± 0.10d	0.99 ± 0.20d	0.74 ± 0.04c	0.80 ± 0.12d
5	0.24 ± 0.05b	0.18 ± 0.00bc	2.19 ± 0.23c	2.39 ± 0.15c	1.52 ± 0.09b	1.49 ± 0.03c
7	0.33 ± 0.00a*	0.24 ± 0.01ab	5.02 ± 0.23b*	4.14 ± 0.31b	2.74 ± 0.32a*	2.00 ± 0.07b
9	0.32 ± 0.05ab	0.27 ± 0.02a	6.18 ± 0.38a	6.67 ± 0.15a	2.92 ± 0.17a	2.83 ± 0.12a

Note: ME: mining ecotype, NME: non-mining ecotype. The data mean the average ± standard error of 3 replicates. Data with different small letters indicate statistically different among growth periods (*p* < 0.05). * represents statistically different between ecotypes (*p* < 0.05).

**Table 2:** P accumulation of *P. hydropiper* grown under 3 mM P supplied as IHP for different growth periods (mg plant^−1^).

Growth period(weeks)	Root		Stem		Leaf	
	ME	NME	ME	NME	ME	NME
3	1.06 ± 0.04b	1.36 ± 0.16c	5.34 ± 0.27d	4.48 ± 0.60d	4.61 ± 0.21d	3.48 ± 0.30d
5	2.18 ± 0.03a*	1.62 ± 0.07bc	15.40 ± 1.80c	15.01 ± 1.32c	9.28 ± 0.30c	7.87 ± 0.08c
7	2.31 ± 0.28a	2.02 ± 0.07ab	31.07 ± 1.59b*	22.79 ± 1.35b	13.42 ± 1.35b*	9.87 ± 0.04b
9	2.35 ± 0.33a	2.28 ± 0.15a	40.94 ± 2.35a*	33.32 ± 0.32a	15.49 ± 0.57a*	11.96 ± 0.03a

Note: ME: mining ecotype, NME: non-mining ecotype. The data mean the average ± standard error of 3 replicates. Data with different small letters indicate statistically different among growth periods (*p* < 0.05). * represents statistically different between ecotypes (*p* < 0.05).

**Table 3:** Biomass of *P. hydropiper* grown under perlite media containing 3 mM P supplied either as G1P, AMP, ATP, IHP, or Pi for 5 weeks (g plant^−1^ DW).

P sources	Root		Stem		Leaf	
	ME	NME	ME	NME	ME	NME
G1P	0.14 ± 0.01b	0.08 ± 0.01b	2.33 ± 0.10c*	1.68 ± 0.17b	0.89 ± 0.06c	0.63 ± 0.09b
AMP	0.12 ± 0.01b	0.07 ± 0.01b	2.33 ± 0.14c*	1.37 ± 0.13b	0.92 ± 0.06c	0.56 ± 0.10b
ATP	0.10 ± 0.02b	0.06 ± 0.01b	2.07 ± 0.27c	1.73 ± 0.15b	0.89 ± 0.13c	0.76 ± 0.06b
IHP	0.31 ± 0.03a	0.35 ± 0.03a	3.36 ± 0.34b	3.12 ± 0.14a	1.72 ± 0.13b	1.94 ± 0.17a
Pi	0.30 ± 0.02a	0.37 ± 0.04a*	4.30 ± 0.32a	3.68 ± 0.27a	2.21 ± 0.17a	2.14 ± 0.08a

Note: ME: mining ecotype, NME: non-mining ecotype. The data mean the average ± standard error of 6 replicates. Means labeled with different small letters are significantly different (*p* < 0.05) among P sources, and * indicates significant difference (*p* < 0.05) between ecotypes.

**Table 4:** P accumulation of *P. hydropiper* grown under perlite media containing 3 mM P supplied either as G1P, AMP, ATP, IHP, or Pi for 5 weeks (mg plant^−1^).

P sources	Root		Stem		Leaf	
	ME	NME	ME	NME	ME	NME
G1P	1.39 ± 0.15b*	0.50 ± 0.13c	14.09 ± 0.24c*	7.92 ± 0.20c	3.64 ± 0.42b	2.81 ± 0.29b
AMP	1.80 ± 0.10b*	0.45 ± 0.15c	10.19 ± 0.76d*	6.01 ± 0.69cd	3.79 ± 0.36b*	1.61 ± 0.18b
ATP	0.50 ± 0.05c	0.55 ± 0.07c	8.16 ± 0.17d*	4.23 ± 0.38d	2.75 ± 0.39b	2.11 ± 0.01b
IHP	1.71 ± 0.21b	1.73 ± 0.34b	21.83 ± 1.93b*	16.85 ± 0.97b	9.48 ± 0.81a	10.14 ± 0.37a
Pi	2.47 ± 0.10a	2.32 ± 0.20a	29.76 ± 0.80a*	22.82 ± 0.46a	10.78 ± 0.77a	11.12 ± 0.47a

Note: ME: mining ecotype, NME: non-mining ecotype. The data mean the average ± standard error of 6 replicates. Means labeled with different letters are significantly different (*p* < 0.05) among P sources, and * indicates significant difference (*p* < 0.05) between ecotypes.
